# Mechanisms of Action of Microbial Biocontrol Agents against *Botrytis cinerea*

**DOI:** 10.3390/jof7121045

**Published:** 2021-12-06

**Authors:** Rocío Roca-Couso, José David Flores-Félix, Raúl Rivas

**Affiliations:** 1 Department of Microbiology and Genetics, Edificio Departamental de Biología, University of Salamanca, 37007 Salamanca, Spain; raulrg@usal.es; 2 Institute for Agribiotechnology Research (CIALE), 37185 Salamanca, Spain; 3 CICS-UBI–Health Sciences Research Centre, University of Beira Interior, 6201-506 Covilhã, Portugal; 4 Associated Unit, University of Salamanca-CSIC (IRNASA), 37008 Salamanca, Spain

**Keywords:** *Botrytis*, biocontrol, mechanisms, Induced systemic resistance, biopesticide, rhizobacteria

## Abstract

*Botrytis cinerea* is a phytopathogenic fungus responsible for economic losses from USD 10 to 100 billion worldwide. It affects more than 1400 plant species, thus becoming one of the main threats to the agriculture systems. The application of fungicides has for years been an efficient way to control this disease. However, fungicides have negative environmental consequences that have changed popular opinion and clarified the need for more sustainable solutions. Biopesticides are products formulated based on microorganisms (bacteria or fungi) with antifungal activity through various mechanisms. This review gathers the most important mechanisms of antifungal activities and the microorganisms that possess them. Among the different modes of action, there are included the production of diffusible molecules, both antimicrobial molecules and siderophores; production of volatile organic compounds; production of hydrolytic enzymes; and other mechanisms, such as the competition and induction of systemic resistance, triggering an interaction at different levels and inhibition based on complex systems for the production of molecules and regulation of crop biology. Such a variety of mechanisms results in a powerful weapon against *B. cinerea*; some of them have been tested and are already used in the agricultural production with satisfactory results.

## 1. Introduction

Agriculture is one of the main human activities at the economic level. It supplies food for a human population that is estimated to grow to reach 9 billion people by 2050 [1,2]. Crop diseases are responsible for extreme economic losses, and they may be caused by different types of organisms [3]. It is estimated that at least 20–40% of these losses are caused by pathogenic infections, and they account for losses of USD 40 billion per year worldwide [4]. In fact, the FAO (Food and Agriculture Organization) estimates that 14% of global crop production losses are due to plant diseases, with fungi accounting for 42% and bacteria for 27% [1].

Indeed, fungi are one of the most important threats to agriculture systems [5,6,7]. This is because they possess characteristics that make them dangerous, such as their high virulence, that is, the relative capacity of a microbe to cause damage to a host [8]. Additionally, fungi are known for their high reproductive potential, elevated dispersion, and ability to disseminate their reproductive forms, resulting in a great number of host individuals being infected in a short period of time [3]. In addition, they have the ability to survive under unfavorable conditions for long time periods because they are spore-forming microorganisms [9].

The most outstanding of all plant pathogenic fungi, which causes economic losses of USD 10 to 100 billion worldwide [10], is *Botrytis cinerea*, known for its ability to expand and colonize different crops [6,11]. Unlike other members of the *Botrytis* genus, it has a broad spectrum of hosts, infecting more than 1400 plant species [10,12]. It mainly uses dicotyledonous hosts, but it also can attack monocotyledonous hosts [13], and it affects a wide range of organs, including flowers, fruits, stems, and leaves [14]. Additionally, it has been reported to infect field- and greenhouse-grown horticultural crops before harvesting [15] and during the post-harvest storage [16]. In fact, *B. cinerea* is the main cause of agriculture losses during the postharvest period because of its unspecific host and the variety of organs it infects [17]. Its high virulence has been recently associated with a long terminal repeat retrotransposon called *Boty* and/or a DNA transposon called *Flipper*. This has resulted in a separation into four genetically different types of *B. cinerea* inside the species: *Boty* or *Fliper*, depending on which element it possesses; *trasposa* if it has both elements; and *vacuma* if it has none of them. The fungi that possess transposable elements are more virulent, and even those belonging to the *Boty* type are able to release small RNAs that knock out some plant defense genes [18].

*B. cinerea,* also known as gray mold [19], is the asexual form (anamorphic) of a necrotrophic fungus whose sexual form (teleomorphic) is called *Botryotinia fuckeliana*, an ascomycete [20]. It is a necrotrophic fungus, meaning that it induces plant cell death to ensure its nutrient supply [6]. Thus, it follows a stationary disease cycle, starting with conidia that are produced during the previous winter, airlifting on the host surface during spring [12,21], and attaching there through physical surface interactions [22]. Firstly, a weak attachment is formed through hydrophobic interactions between the host and conidial surfaces [23], followed by stronger binding resulting from conidia germination and the production of germ tubes, whose extracellular matrix acts as an adhesive to the host surface [22,24]. Conidial germination is dependent on several factors, with high humidity (>93%) [25] and nutrient availability being the most important [12,26]. Additionally, some gases, such as ethylene, have been proven to induce germination [27,28]. After conidia germination, *B. cinerea* can penetrate tissues through active or passive invasion. This means that the fungus can take advantage of previous wounds and stomata for penetration or can directly attack healthy tissues [22]. At this level, invasion induces programmed cell death, a typical defense response against pathogens [29]. This is the consequence of the production of diffusible factors with phytotoxic activity, such as toxins, oxalic acid, and reactive oxygen species (ROS), which penetrate plant cells [30,31], allowing *Botrytis* to penetrate plant barriers [15]. Infection then expands to surrounding cells by degradation of the cell wall, leveraging the nutrients resulting from the process [32]. Finally, expansion continues until plant defenses have broken down, and *Botrytis* experiences vigorous growth, spreading disease [19] and resulting in plant maceration, fungal sporulation, and the production of new conidium [22].

Traditionally, chemical pesticides have been used for *B. cinerea* control [33]. However, in recent years, many arguments against their use have been raised [17]. These have mostly concerned environmental damage since pesticides accumulate in soils as toxic residues, as well as the development of resistance [16,34,35] resulting from pesticide overuse [36], and single-site fungicide use, which enhances the development of specific resistance [37]. This occurs because *B. cinerea* has a very short life cycle, a high reproductive tax, and high genetic variability, which make it a high-risk pathogen for the development of fungicide resistance [38].

Thus, biopesticides have been proposed as a potential alternative to treat this pathogen. Although there is no common definition for biopesticides, they can be described as plant pest control products manufactured from living microorganisms [39]. In recent years, they have achieved recognition because they present some positive characteristics, such as their higher selectiveness and their lower manufacturing costs [40]. Environmentally, they are also more efficient because their use does not release toxic compounds, and it decreases the negative effects of plant pathogens and increases positive responses by the plants [41]. Additionally, they usually have several modes of action, thus reducing the development of resistance [37], which is a key factor in *Botrytis* control owing to the quick development of resistance by this pathogen [38]. Unfortunately, biopesticides come with some disadvantages, such as a lower rate of kill compared with chemical pesticides, and they are negatively influenced by environmental conditions [40]. Therefore, another approach to pest control has been taken, which is the combination of biopesticides with chemicals, allowing maintenance or improvement of the control effect through a 10-fold reduction in the amount of pesticides and a reduction or elimination of residues [42]. This could also be a desirable strategy against the frequent appearance of strains resistant to numerous commercial chemical fungicides [43,44], which limits their implementation at a practical level [42].

Of the many different microorganisms, one type has been highly studied in the development of new biopesticides: plant-associated microorganisms, which are able to act as biopesticides and enhance plant growth at the same time [35,45]. These microorganisms are known as Plant Growth Promoting (PGP), and they are microbes living in the plant environment (inside plants’ rhizosphere or phyllosphere) with the ability to improve plant development through different mechanisms [46]. It is known that endophytic populations usually come from rhizosphere populations, meaning that plants can establish associations with surrounding microorganisms without being harmed [47]. Additionally, they usually provide some kind of benefit to the plant, for example, through the production of phytohormones, such as auxins, cytokinins, and gibberellic acids [48]; the enhancement of uptake of essential elements, such as iron or phosphorus [49]; and the protection of plants against crop diseases [50]. Within the PGP microorganism family, endophytic microorganisms are those that inhabit the interior of plants, where they can contribute to the host’s growth without causing disease [49]. These have been increasingly recognized because biocontrol efficiency is associated with important limiting factors—niche adaptation and microbial competition [1]. By using endophytes as biocontrol agents, these difficulties can be avoided since these organisms are already adapted to the target plant [51]. Endophytic populations vary from plant to plant and from species to species, and, within the same species, populations not only vary from region to region but also differ with changes in climatic conditions in the same region [49]. A great number of microorganisms have been described; however, most of them have only been tested under laboratory conditions. Thus, an analysis of in-vivo studies considering plant microorganism interactions is essential since the ideal conditions found in the laboratory are far from the reality in the field, where the development of beneficial microorganisms is affected by environmental issues, both biological and nutritional [52].

Several rhizospheric and endophytic microorganisms have been studied with regard to the development of new biopesticides, and currently, some of them are used for *Botrytis* control in crops where, owing to the impact, economic relevance, and intrinsic characteristics of this fungal phytopathogen, the use of biopesticides is a suitable tool for control that improves the sustainability of crop management [17]. Among the biopesticides, there are several species of bacteria, filamentous fungi, and yeasts that are already known to have antifungal activity [53,54], such as *Bacillus subtilis* and *Trichoderma harzianum* [38].

Among the bacteria in this group, the *Bacillus* genus is one of the most studied genera, mainly regarding its production of antibiotic substances, such as bacteriocins and peptide antibiotics, whose targets are closely related bacteria or subtilin, one of the best-characterized antibiotics produced by *B. subtilis* [37,55]. Species of this genus are considered biologically safe, and they are widely used in agriculture [56] because they possess several advantageous properties, such as spore formation, which allows them to survive under unfavorable conditions, for example, heat and drought conditions. Thus, they are highly desirable for use in industry production and storage, as these conditions do not jeopardize the viability and therefore the efficacy of the treatment [54]. Therefore, the *Bacillus* genus is considered one of most effective bacterial genera against phytopathogens [57]. However, there are other genera, such as *Pseudomonas* and *Paenibacillus*, which have also been well-studied in regard to *Botrytis* biocontrol [58]. For example, *Pseudomonas aeruginosa* has been well-studied for its strong antagonistic effect against *B. cinerea* [59]. *Paenibacillus* as well as *Bacillus* is known to produce a large number of secondary metabolites, such as lipopeptide antibiotics, antifungal proteins, volatile compounds, and lytic enzymes [38]. In fact, it was originally included within *Bacillus* but later reclassified as a separate genus [60].

Regarding fungi, interest in them has increased because they possess biological characteristics that make them a suitable option; these include their high reproductive rate, both sexually and asexually; their short generation time; and their target-specific characteristic [61]. One of the most studied fungi is the *Trichoderma* genus owing to its multiple mechanisms of action, such as its induction of plant resistance, mycoparasitism, antibiosis, and competition for space and nutrients [37]. In fact, the first fungus registered as a biocontrol agent for plant disease with the United States EPA was *T. harzianum* ATCC 20476 in 1989. [1]. Other filamentous fungi, such as *Gliocladium*, are also known for their activity against *Botrytis* [58] and can directly attack the fungi through aggressive hyphal puncturing of cell walls [1]. Fungi produce several secondary metabolites with biocontrol interest or another biotechnological purposes, for example, biofuel production or commercial drugs [41].

Finally, yeasts have also been studied as biocontrol agents, mainly in fruits [7,62]. Yeasts present some advantages over bacteria, such as their simple nutritional requirements, their ability to colonize dry surfaces for long periods of time, and their fast growth [63]. They do not produce toxic metabolites; avoid negative environmental or toxicological impacts [64], unlike some bacteria [10,19]; and possess many different modes of action, including competition for nutrients and space, the production of toxins, enzyme secretion, the production of volatile organic compounds (VOCs), parasitism, and the induction of systemic resistance [65]. Some examples of yeasts with antagonistic activity against *B. cinerea* are *Candida oleophila* in apples after harvest [66], *Pichia guilliermondii* in tomato fruits, and *Candida sake* against major post-harvest apple pathogens [67].

*B. cinerea* is difficult to control because of its variety of attack modes, its ability to infect diverse hosts, and the ability of both its sexual and asexual stages to survive for extended periods under unfavorable conditions [10]. In this sense, the success of one single method is not likely, and 1 to 20 different methods are usually needed during one season [68]. Thus, knowing the mechanisms of action by which each microorganism interacts with pathogens is a key factor in the development of new biopesticides [38]. In conclusion, studying and understanding different modes of action are necessary to develop appropriate biopesticides [64].

**Figure 1:**
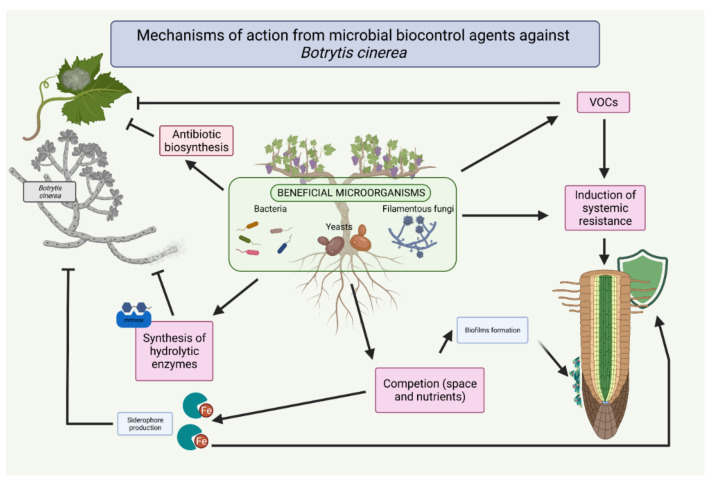
Mechanisms of action from microbial biocontrol agents against *Botrytis cinerea*.

In this review, we looked at the different mechanisms of action used for the biocontrol of the pathogen *B. cinerea* by both bacterial and fungal agents (Figure 1).

## 2. Production of Diffusible Molecules

Microorganisms can act against Botrytis by producing secondary metabolites that defend through the medium and reach the pathogen [69]. There are several antifungal molecule types, and in this review, we looked at two of the most important groups, as summarized in Table 1.

### 2.1. Antimicrobial Molecules

These are microbial compounds synthetized by microorganisms (bacteria or fungi) that, at low concentrations, are able to kill or inhibit the growth of other microorganisms [2,89]. They encompass a chemically heterogeneous group of organic, low-molecular-weight compounds produced by microorganisms that negatively affect the growth or metabolic activities of other microorganisms [38].

Regarding bacteria as antibiotic producers, some genera have been studied for their efficiency to act against *B. cinerea*, such as *Bacillus* and *Pseudomonas* [53]. On one hand, the genus *Bacillus* is known as one of the most efficient biocontrol agents against plant pathogens [90], as it produces important antimicrobial compounds, such as cyclic lipopeptides (LPs) [91]. These molecules are low-molecular-weight, cyclic, amphiphilic oligopeptides that are synthesized by enzyme complexes called Non-Ribosomal Peptide Synthetases (NRPSs) [56], and they have recently attracted attention for their broad-spectrum activity against plant pathogens [92]. They can be classified into three groups based on their amino acid sequence, with iturin and fengycin highlighted by their surfactant properties [38]. Additionally, other lipopeptide families, such as kurstakin, maltacines, and polymyxins, have been identified in *Bacillus* [93]. Lipopeptides are able to bind to the lipid membrane in cells, increasing their permeability and producing structural damage [38]. In particular, fengycin and iturin open pores in the plasma membrane [94], and it has been reported that they may damage the fungal hypha [95] and permeabilize fungal spores, thus inhibiting their germination [96].

As an example of *Botrytis* biocontrol mediated by *Bacillus* lipoproteins (LP), *Bacillus* sp. XT1 is able to inhibit the growth of the phytopathogen both in vitro and in vivo. In the first case, inhibition rates of 72, 48, 30, and 19% of the mycelium diameter were found after the application of 10, 6, 4, and 2 mg mL^−1^, respectively, on lipopeptides. In-vivo assays developed in grapes, strawberries, and tomatoes infected with *B. cinerea* experienced a disease reduction in fruit treated with XT1 lipopeptides of 100, 12, and 50%, respectively, after *Bacillus* sp. XT1 lipopeptide treatment [56]. Another strain, *B. subtilis* NCD-2, has been shown to produce several secondary metabolites. Some of them, such as surfactin and fengycin, have antifungal activity against *B. cinerea* [72]. Surfactin is another LP with broad-spectrum antimicrobial activity [97]. However, lipoproteins are not the only antifungal metabolites produced by *Bacillus*; there are plenty of others, such as phthalic acid, hept-3-yl isobutyl ester, and propanoic acid, 2-hydroxy-, and methyl ester, produced by *B. amyloliquefaciens* VB7, which showed inhibition rates of up to 46% against *B. cinerea* [98]. On the other hand, *Pseudomonas* is also a genus that may control *B. cinerea* efficiently, since its members produce a wide range of metabolites [38], including 2,4-diacetylphloroglucinol (DAPG), pyrrolnitrin, and phenazine [2]. Another example is *Paraburkholderia phytofirmans* PsJN (previously classified as *Pseudomonas sp.* PsJN), which inhibits *B. cinerea* growth when both microorganisms are grown in the same plate. After microscopic observation, cytoplasm coagulation of *B. cinerea* mycelium was observed [71,99]. This produced mycelium degradation, fungal suppression, and hyphae structure modification [100].

Additionally, there are other bacterial genera that produce secondary metabolites with antifungal activity, for example, *Actinoalloteichus cyanogriseus* 12A22, which produces 2-hydroxyethyl-3-methyl-1,4-naphthoquinone, a compound whose inhibition of *Botrytis* is greater than that produced by amphotericin B, a wide-ranging antifungal compound [70]. Another example is *Ochrobactrum ciceri* MM17, which produces many different metabolites with antifungal activity, both diffusible and volatiles, such as propanoic acid, −hydroxy-methyl ester, phthalic acid, hex-3-yl isobutyl ester and phthalic acid, hept-3-yl isobutyl ester (diffusible), and dimethyl trisulfide and pentadecane (VOC). These bacteria have also been tested against *Botrytis*, showing suppression of 77% of leaf blight [75]. *Pantoea* sp. MQT16M1 is also a great producer of antifungal metabolites, such as salicylamide, maculosin, and herniarin, allowing it to inhibit up to 80% of *Botrytis* mycelial growth [76]

Fungi can also produce antimicrobial molecules [2]. Some examples of such fungi are *Nigrospora oryzae* 2693 and *N. oryzae* 2778, *Trichoderma asperellum* 2739, *Penicillium commune* 2748, *Fusarium proliferatum* 2751, and *Chaetomium globosum* 2773, whose secondary metabolites produced inhibition of 17, 57, 58, 27, 69, and 56% of *Botrytis*, respectively [84]. Some antifungal molecules have been isolated and identified; for example, gliotoxin is produced by some species, such as *Trichoderma* sp. BV1 and *Aspergillus fumigatus* AF293, and it has antifungal activity related to DNA disruption [82,83]. *Gliocladium virens* 41 also produces gliotoxin, which has an inhibitory effect on *B. cinerea* spores germination [101]. Glucose oxidase is another secondary metabolite produced by *T. atroviride* SJ3-4 that also affects spore germination in *B. cinerea,* reducing it by up to 61% [86]. Indeed, *Trichoderma* produces several metabolites with antifungal activity that may be efficient for *B. cinerea* biocontrol, like dermadin, trichovirdin and sesquiterpene, heptalic acid [88]. The use of *T. harzianum* T-39 has also been effective in controlling infection in tomato plants in combination with the fungicide dicarboximide in a single application, reducing by half the treatments necessary to control the infection by *B. cinerea* [14].

In contrast, yeasts do not produce as many secondary metabolites as bacteria or filamentous fungi. In this sense, little is known about this mode of action in yeast. However, *Aureobasidium* sp. JYC1525, *Saccharomyces cerevisiae* JYC137, and *Candida stellimalicola* JYC2120, have been shown to reduce *B. cinerea* mycelial growth by 36.5, 26.8, and 14.5% respectively, by secreting diffusible compounds [19], and some metabolites have been studied and highlighted for their biocontrol activity [65]. Some of the most important of these are protein-like killer toxins, which are antimicrobial metabolites secreted by yeasts [86]. The first report of this activity was in *S. cerevisiae* CBS8112, but few yeasts have been shown to secrete killer toxins since then [88]. Additionally, *Pichia membranifaciens* CYC 1106 is able to produce killer toxins and has been studied for its biocontrol activity against *B. cinerea* [86]. Additionally, some other secondary metabolites with general antifungal activity have been described [65], such as aureobasidins, liamocins, 2-propylacrylic acid, and 2-methylenesuccinic acid, which are produced by *Aureobasidium pullulans* L47 [85].

### 2.2. Siderophores

Another large group of diffusible molecules with antifungal activity is the siderophores. These are low-molecular-weight organic molecules (200–2000 Da) that are involved in microbial iron metabolism [102]. Iron is an essential micronutrient that is present in a high percentage in soils. However, this element has extremely low solubility in common soil conditions (pH > 6), making its acquisition hard for living beings [52]. In this sense, iron bioavailability has become a limiting factor that may lead to competition among organisms. Siderophores are molecules with a high affinity for iron, allowing microorganisms to obtain this element [103]. Siderophores have been studied for their applications in a variety of fields, such as environmental science and medicine [104]. Regarding agriculture, they are not only used for biocontrol but also for soil fertility improvement. Biocontrol is achieved through iron sequestration. Siderophores are secreted and, under low-iron conditions, they form a ferric–siderophore complex that avoids acquisition by other microorganisms, reducing the availability of this micronutrient [105]. Under iron-deficient conditions, siderophore production may be a useful tool for phytopathogen inhibition in the host [103]. *B. cinerea* needs iron for growth. If iron availability is restricted, their spore germination rate and mycelial growth will decrease [106]. Some specific siderophores have been proven to have activity against these fungi, and they have been widely studied [38].

There are three main siderophore types, and according to which part of the molecule chelates the iron, they are classified into hydroxymates, catecholates, and carboxylates [107]. Some authors consider there are two other groups of siderophores: phenolates, which are included in the catecholate group, and mixed siderophores [108].

Hydroxymates are the most common siderophores [109]. They are produced by both bacteria and fungi, and their chelation site is a hydroxymate group (-CO-N(O–)-) that is synthetized from either lysine or ornithine. Lysine-derived examples are aerobactin, which is typically produced by *Escherichia coli*, and mycobactin, which is produced by *Mycobacterium* spp. [110]. On the other hand, ornithine derivatives include pyoverdine, known as a typical *Pseudomonas* siderophore; exochelin, which is a characteristic of *Mycobacterium* spp.; and ornibactin, which is also produced by some strains of *Pseudomonas* strain CHA0 [50]. Indeed, this genus produces a great number of secondary metabolites with antifungal activity against *B. cinerea*, for example, *P. fluorescens* fp-5, which prevents plant infection after preharvest treatment thanks to its hydroxamate-type siderophores [78]. There are several examples of this type of siderophore with antifungal activity, such as pyoverdine or pseudobactin, which are both produced by *Pseudomonas* strain CHA0 and are able to suppress *Fusarium oxysporum* [50]. Fungi produce additional hydroxymate siderophores, like ferrichrome and fusarinine, produced by *Fusarium* spp., and coprogens, produced by *Trichoderma* spp. [111]. In fact, fungal siderophores mainly belong to this group, and they can be divided into three families according to their structure [112]. Fusarinines are either monomers, linear dimers or trimers, or cyclic trimers; coprogens are linear dihydroxamate and trihydroxamate ligands composed of fusarinine units and ferrichromes, which are cyclic hexapeptides [113,114,115]. Some of these fungal siderophores have been related to antifungal activity against other species [116]. For example, *Acremonium* spp. are endophyte fungi with siderophore activity, and some species, such as *A. persicinum* MF-347833, have shown antifungal effects against some phytopathogens, like *B*. *cinerea* [80,117,118]. Among yeasts, iron is also an essential nutrient, meaning iron depletion is a well-known mode of action against fungi [65]. An example from the hydroxymate group is rhodotorulic acid, a siderophore produced by *Rhodotorula glutinis* ySL 30 with activity against *B. cinerea*, which has been shown to delay spore germination and reduce apple decay by 72% [87,119]. Another example is *Metschnikowia pulcherrima* MPR3, which has been shown to control *Botrytis* in vivo and is associated with iron sequestration [64] mediated by the siderophore pulcherrimin, known for its reddish coloring [120], which is widely produced among yeasts [121] However, pulcherrimin is not always considered a siderophore since it is not a diffusible compound [121].

The second group is catecholates, which are produced exclusively by bacteria. They possess a mono-, di-, or C_6_H_5_OH-hydroxybenzoic acid group that chelates the iron [107,122]. The latter includes phenolates, which are mostly produced by enterobacteria [123]. Some of the most well-known members are enterobactin produced by *E. coli*, pyochelin produced by *P. aeruginosa,* salmochelin produced by *Salmonella enterica*, bacillibactin produced by *Bacillus* spp., agrobactin produced by *Agrobacterium tumefaciens*, parabactin produced by *Paracoccus denitrificans*, and azotobactin produced by *Azotobacter vinelandii* [107,124]. Members of *Bacillus* have been studied for their ability to produce siderophore-mediated antifungal activity by producing bacillibactin, which inhibits fungal growth against some pathogenic fungi, such as *Macrophomina phaseolina*, *Fusarium moniliforme,* and *B. cinerea* [73,125,126,127]. Moreover, *P. aeruginosa* 7NSK has a protective effect against *B. cinerea* when pyochelin and pyocyanin are produced together and when pyoverdine is produced, in tomato plants and *Arabidopsis thaliana*, respectively [77,128]. Additionally, siderophore production by other bacteria has been associated with antifungal activity against *B. cinerea*, for example, *Kosakonia radicincitans*, which produces enterochelin. In addition to iron sequestration, siderophores can achieve biocontrol through other pathways. For example, enterochelin, produced by the enterobacterium *Rahnella aquatilis* BNM, has been shown to inhibit *B. cinerea* by blocking polygalacturonase, a cell-wall-degrading enzyme that is involved in the colonization of host tissues [79]. Similar activity was shown when enterochelin produced by *K. radicincitans* DSM 16656 was tested against *B. cinerea* in apple fruits, reaching a decay reduction of 52% [74]. Finally, *Acinetobacter calcoaceticus* HIRFA32 and *Pseudomonas fluorescens* Mst 8.2 produce a catechol-type siderophore that inhibits fungal mycelial growth by 46.9% in vitro and 71.5% in planta [129,130].

Finally, carboxylates are only produced by a few bacteria, like certain *Rhizobium* spp. and *Staphylococcus* spp., and by fungi belonging to *Mucorales* [111]. Here, the chelation group is either a hydroxyl or carboxyl. Rhizobactin, produced by *Rhizobium* [131], and staphyloferrin, produced by *Staphylococcus* spp. [132], are highlighted in this group. Rhizobactin is also produced by some Zygomycetes fungi, it being the only group where fungal siderophores can be found, along with hydroxamates [112]. It has been related to biocontrol activity against some plant pathogens both in vitro and in vivo [133].

Additionally, siderophores have been also studied for their ability to stimulate plant-induced systemic resistance (ISR) in plants [38], making them more resistant to pathogens by inducing physiological changes throughout the entire plant [134]. This has been shown in studies of different bacteria, and siderophores from *Pseudomonas* have gained importance for their high iron affinity. Thus, pyoverdine and pyochelin have been studied against phytopathogenic fungi, such as *B. cinerea* [135]. These siderophores are highly related to ISR since it has been proven that repression of their synthesis increases plant susceptibility to *B. cinerea* [128]. In the same way, it has been found that *P. syringae* pv. *tomato* can trigger ISR in *A. thaliana* through production of the siderophore pseudobactin, and this has also been proven to positively influence *B. cinerea* disease reduction [136]. Therefore, determining how siderophores promote ISR remains poorly understood.

## 3. Synthesis of Volatile Organic Compounds

Volatile Organic Compounds (VOCs) are usually small, odorous compounds (<C15) of low molecular mass (<300 Da) with a high vapor pressure, low boiling point, and a lipophilic moiety. They belong to different chemical classes, such as terpenes and alcohols [137]. Some of them are known to interact with other microorganisms, and since they can travel long distances, they are considered good mechanisms of action for biocontrol [69]. However, these molecules have not received as much attention as other antagonistic mechanisms [103]. These molecules present some advantages over other mechanisms. For example, they are effective in low concentrations, they diffuse through air-filled pores in soil, and they can act on pathogens without establishing actual physical contact with them [88,103]. Additionally, they can also promote plant growth, enhance plant tolerance to abiotic stress, and elicit induced systemic resistance (ISR) [37].

Among bacteria, those in the genus *Bacillus* are known as great producers of secondary metabolites, including VOCs [103]. Some have been studied for their action against *B. cinerea*. Thus, *Bacillus velezensis* ZSY-1 has been identified as a producer of several volatile compounds, such as pyrazine (2,5-dimethyl), benzothiazole, phenol (4-chloro-3-methyl), and phenol-2,4-bis (1,1-dimethylethyl), whose inhibition rates against *B. cinerea* in vitro were found to be 100, 100, 100, and 91.19%, respectively [138]. Recently, other members of the *Bacillus* genus, including *B. nakamurai* TR2, *B. pseudomycoides* DHT2, *B. proteolyticus* H2F1, and *B. thuringiensis* H1R2, were found to produce VOCs against *B. cinerea*, such as 3-methylbutan-1-ol, sulfur-containing compounds, 2-heptanone, and dodecanal [139]. Additionally, the metabolome can be influenced by the presence of this pathogen. In this sense, *B. amyloliquefaciens* VB 7 produces extra VOCs, such as oxirane, 3,5-octadiyne, and formic acid, when it is co-cultured with *B. cinerea* biomass. This can be related to the antifungal activity shown both in vitro and in planta, which inhibited 46% of fungi [98].

In the same way, *Pseudomonas* spp. are also great metabolite producers [139]. For example, *P. chlororaphis* ZL3 was evaluated for its antifungal activity, and 23 VOCs were found [140]. In the same way, *P. protegens* CHAo was evaluated for inhibition of several fungi, being positive for those related to *B. cinerea*, because of the production of two volatile molecules, ammonia and dimethyl trisulfide [141]. Most experiments of this type have been performed in vitro. For example, *P. aeruginosa* LV, which produces phenazine-1-carboxylic acid, was shown to reduce *Botrytis* mycelial growth by 50% [142]. However, some in-vivo assays were also done. For example, the antifungal activity of *P. fluorescens* ZX due to VOCs was evaluated, showing that dimethyl trisulfide and geranyl formate treatment resulted in complete inhibition of the fungi [143].

Additionally, bacteria other than *Bacillus* and *Pseudomonas* have been studied for their antifungal VOC production. For example, *Streptomyces* sp. S97 was shown to inhibit 87% of *B. cinerea* symptoms resulting from the production of VOCs, mainly 3-carene 2,5-dione, geosmin, beta-cubebene, and one phenolic compound [144]. *Pantoea* sp. MQT16M1 was also studied for its antifungal activity against *Botrytis* and was shown to reduce mycelial growth reduction by 90%, owing to the production of VOCs, such as phenylethyl alcohol [145,146]. Finally, lactic acid bacteria like *Lactiplantibacillus plantarum* UFG 121 have also been shown to have antifungal activity against *B. cinerea*, reducing its concentration by 40–80%, and this has been associated with a pH reduction, probably due to the production of phenyllactic acid and 4-hydroxyphenyllactic acid [147,148].

Fungi are also producers of VOCs, and they have been studied for their biocontrol activity against *Botrytis*. Some, like *Hypoxylon* sp. CI-4, an endophytic fungus isolated from *Persea indica* that produces 1,8-cineole and 1-methyl-1,4-cyclohexadiene, and alpha-methylene-alpha-fenchocamphorone, have been tentatively identified among many others. *Hypoxylon* sp. CI-4 is known for displaying maximal VOC antimicrobial activity against *Botrytis*, resulting in 100% inhibition [149]. *M. anisopliae* Ma70 has been shown to have antifungal activity against *B. cinerea* in vitro owing to the production of VOCs. Forty-one volatile compounds have been isolated, and one of them, 1-octanol, has been reported as an effective treatment for biocontrol since it inhibits conidia germination and mycelium growth of *B. cinerea* [150]. An unusual strain of *Phomopsis* sp. By254, isolated from *Odontoglossum* spp. (*Orchidaceae*), produces a mix of gases with antifungal properties against a wide range of plant pathogenic test fungi, including *Botrytis* [151]. Some VOCs have been identified, such as sabinene, 1-butanol, 3-methyl, benzeneethanol, 1-propanol, 2-methyl, and 2-propanone [152]. Finally, *Trichoderma* has also been studied for its volatilome, which has an antifungal effect, and some effective compounds, such as trichodermol, harzianum A, and harzianolide, have been discovered [101].

Regarding yeasts, there are also many species that show antifungal mechanisms of this type. Several yeasts, mostly food yeasts, such as *W. anomalus*, *M. pulcherrima*, *S. cerevisiae,* and *A. pullulans* EXF-6519, have VOC-derived antifungal activity against *B. cinerea,* with 3-methyl-1-butanol being the most effective compound [65,153]. Some of those VOCs have been identified, such as 2-ethyl-1-hexanol, which is produced by *Sporidiobolus pararoseus* YCXT3 and inhibits spore germination and mycelial growth [154], or 1, 3, 5, 7-cyclooctatetraene, 3-methyl- 1-butanol, 2-nonanone, and phenylethyl alcohol, which are produced by *C. intermedia* C410 [155]. Additionally, two of the most recently discovered microorganisms are *Scheffersomyces spartinae* W9 and *Candida pseudolambica* W16, which are VOC producers with *B. cinerea* antifungal activity, both in vitro and in planta [156].

Nowadays, hundreds of microorganisms have been described as VOC producers, and uncountable compounds have been isolated (Table 2) [157]. As previously stated, some VOCs have antifungal activity; however, the inhibitory effect is not always the same, as it depends on the specific relationship between the producer and the target [38]. Additionally, the quantity and diversity of volatile compounds produced by a specific microorganism vary depending on several factors, such as the availability of nutrients and oxygen [158]. In this sense, it can be concluded that VOCs and their effects cannot be associated with specific microbes, and their use as biofungicides must be specifically studied [159].

## 4. Hydrolytic Enzymes

Another type of molecule that is highly involved in *Botrytis* biocontrol is the hydrolytic enzyme. These are able to cleave polymeric compounds, such as chitin, proteins, cellulose, hemicellulose, and even DNA [89], and they also can interfere with pathogen metabolic activity [160], inhibit conidia germination, and lyse germ tubes [58].

There is no specific composition for fungal cell walls, but most have a layered structure. While the innermost layer is relatively conserved, the outer layers are more heterogeneous [161]. In most fungal species, the inner cell wall composition is usually a core of covalently attached, branched β-[1,3] glucan and chitin [162]. This configuration is responsible for maintaining cell integration [161]. Biochemical analyses of *B. cinerea* cell walls have shown that they mainly constitute neutral sugars and proteins, with glucose, arabinose, galactose, xylose, and mannose being the most common neutral sugars present. Additionally, chitin and uronic acids have been detected [163]. In fact, microorganisms with the ability to cleave chitin are already used for the control of microbial pathogens and insect pests [34]. In summary, the main fungal cell wall components are chitin (around 20%), glucans (between 50 and 60%), and proteins (between 20 and 30%) [103].

Therefore, glucan is the main constituent of *Botrytis* cell walls, and its degradation may result in its inhibition. This carbohydrate can be hydrolyzed by two main enzymes, exo-β-1,3-glucanase and endo-β-1,3-glucanase. The first is able to cleave glucose residuals from the non-reducer extreme, while the second acts in the bonds to aleatory sites along the polysaccharide chain [103]. Additionally, chitin hydrolyzation is a good biocontrol mechanism. Chitin is a non-branched homopolymeric N-acetyl glucosamine with 1,4 bonds [164]. These links can be cleaved by two possible enzymes, exo-chitinase or N-acetyl-b-glucosaminidase, which hydrolyzes the NAG extreme residues, and endo-chitinase, which randomly breaks link sites along the polymer chain [103]. Disruption of the main cell wall components may induce fungal suppression, so microorganisms that are able to produce related lytic enzymes are good candidates for biopesticide development (Table 3) [38].

The genera *Bacillus* and *Pseudomonas* are considered to be two of the most efficient antagonists in phytopathogen control owing to the direct action of chitinase [103]. Regarding the genus *Bacillus*, studies on *B. halotolerans* KLBC XJ-5 have shown that this strain undergoes chitinase and β-1,3-glucanase secretion, and this has been linked with its ability to reduce *B. cinerea* mycelial growth and conidial germination [166]. In the same way, *Bacillus amyloliquefaciens* Y1 antifungal activity has been related to the production of hydrolytic enzymes, such as β-1,3-glucanase [165]. *Bacillus subtilis* KLBC BS6 has also been shown to exhibit antifungal activity against *B. cinerea* through several mechanisms, including chitinase production [167]. Additionally, *Paenibacillus xylanexedens* Z2–4 is a chitinase producer with antifungal activity against several pathogens, such as *B. cinerea* [168]. On the other hand, *Pseudomonas* spp. are also interesting. For example, *P. elgii* HOA73 possesses strong chitinolytic activity, which is associated with the complete suppression of spore germination of *B. cinerea* in vitro [34].

Species, including those in other genera, such as *Virgibacillus marismortui* M3-23, *Terribacillus halophilus* J31, *Halomonas elongata* L80, *Planococcus rifietoensis* M2-26, *Staphylococcus equorum* B1-35, and *Staphylococcus sp.* J23, which produce chitinase, β-1,3-glucanase, cellulase, and protease, have also shown in-vitro *Botrytis* antifungal activity related to the production of hydrolytic enzymes. They reduce the concentrations of these enzymes by 50 to 92% in infected fruits [38,170]. These activities have also been studied in planta in some species. For example, *Serratia plymuthica* C48, which inhibits spore germination and germ-tube elongation, is a high chitinolytic enzyme producer. Studies performed on this strain have purified two main enzymes, CHIT60 and CHIT100, and these have been tested for their potential use in *Botrytis* biocontrol by analyzing spore germination and germ-tube elongation. Results showed reductions of 28 and 31.6%, respectively, when applying CHIT60 and reductions of 78 and 63.9%, respectively, when applying CHIT100 [169].

Regarding fungal agents that act against *Botrytis*, one of the most well-known agents of *Botrytis* control is T39 of *T. harzianum*, which is able to produce proteases that hydrolase some essential *Botrytis* enzymes, like exo- and endo-polygalacturonase, pectin methyl esterase, pectate lyase, cutinase, chitinase, and β-1,3-glucanase, reducing spore germination [175]. Extracellular proteases are also produced by *Trichoderma* spp., and they have recently received more attention since they play an important role in acting against phytopathogens. They have been tested against *B. cinerea* and found to act in different ways. They can inactivate the fungal hydrolytic enzymes needed for infection, they are useful for protein competition, and they can also directly attack components of fungal cell walls, facilitating cell disruption [174,179]. This is not the only species with this capacity; several additional species have been tested for their biocontrol in vivo [180]. Some fungi, like *Gliocladium*, have been highlighted for their production of hydrolytic enzymes, like endochitinase in *G. virens* 41 or β l-3 glucanase and chitinase produced by *G. roseum* Bainier, resulting in a reduction of *Botrytis* growth of up to 90% [172,173]. Additionally, new endophytes are being discovered; *Albifimbria verrucaria* SYE-1 was recently isolated from grape leaves, and chinolytic activity against *B. cinerea* has been reported [171].

Finally, yeasts also secrete lytic enzymes, such as chitinases, proteases, and glucanases, which are involved in biocontrol activity. As previously stated, chitin is a key factor in fungal cell wall degradation, and in this sense, some genera, such as *Aureobasidium, Candida, Debaryomyces, Metschnikowia, Meyerozyma, Pichia, Saccharomyces, Tilletiopsis*, and *Wickerhamomyces*, have been studied for their chitinolytic activity [65]. Specifically, *Galactomyces candidum* JYC1146 and *Aureobasidium* sp. JYC1525 have shown chitinolytic activity that may be involved in *Botrytis* biocontrol [19]. However, *Aureobasidium pullulans* PI1 has also been studied for its ability to excrete β-1,3-glucanase, pectinase, and protease to the medium. While β-1,3-glucanase and protease enzymes act directly on the *Botrytis* cell wall, pectinase has the ability to penetrate and colonize plant cell walls, thus enhancing its ability to compete with pathogens for nutrients [63]. Indeed, glucanase activity against *Botrytis* has been well-studied in yeasts. For example, two genes, PaEXG1 and PaEXG2, encoding for exo-β-glucanases in *Pichia anomala* K, are responsible for antifungal activity against *B. cinerea* [177]. Finally, proteases are not as well-studied as other microorganisms since they are only produced during the later growth stages [65]. Despite this, some alkaline serine proteases produced by *A. pullulans* PL5 can reduce spore germination and the germ-tube length of *B. cinerea* in vitro [176]. It has also been found that the use of the β-1,3-glucanase-producing *Cryptococcus laurentii* strain LS28 in combination with different antifungals from the benzimidazoles and thiabendazoles family allows postharvest control of the incidence of strains resistant and nonresistant to *B. cinerea* [178].

## 5. Other Mechanisms

In this review, we summarized antifungal activities that depend on secondary metabolite production. However, there are mechanisms of great interest where microorganisms play major roles (Table 4).

### 5.1. Competition

*B. cinerea* is a necrotrophic pathogen, meaning it obtains organic nutrients from dead cells that it has already killed [194]. In this way, necrotrophic fungi need exogenous nutrients for germination and for growth on plant surfaces in a pre-penetration state [14].

Competition for both nutrients and space is a key factor since colonization is only possible when colonizers can obtain the proper amount of nutrients [89]. Previous studies have shown that non-pathogenic microorganisms are able to colonize plant surfaces, thereby limiting the amount of nutrients available, reducing the pathogen spore germination percentage, and thus reducing the host invasion capacity [103]. Additionally, reduction of the amount of nutrients usually reduces germ-tube growth, reducing infection, necrosis, and expansion of the fungi [58].

*Botrytis* biocontrol by competition seems to be efficient since conidial germination, germ-tube growth, and complete infection cannot be completed without a sufficient amount of nutrients [192]. Competition seems to be an effective method for postharvest infections by *B. cinerea* as well [38]. Some bacteria have the ability to compete, and as always, *Bacillus* spp. and *Pseudomonas* spp. are two of the most important genera when it comes to studying biocontrol. Species such as *P. syringae* L-59-66, which have antifungal activity mainly because of competition for nutrients [188], and *B. amyloliquefaciens* BBC047, which is found in tomato leaves where it forms biofilms [181], are good candidates for *B. cinerea* biocontrol. Other species, like *Pantoea ananatis* BLBT1-08 and *Lactobacillus plantarum* CM-3, have been well-studied because they rapidly colonize plant wounds before the establishment of *B. cinerea*, thereby suppressing mycelial growth and disease symptoms [184,185].

Although there are several examples of bacteria that act as competitors, fungi are the most efficient microorganisms for this task since they grow extremely fast, depleting the amount of nutrients available. Two have shown the best results under laboratory and field conditions: *T. harzianum* T39 and *Ulocladium atrum* 385 [58]. Moreover, *Aureobasidium pullulans* L1, which competes for space and amino acids [105], *Chlonostachys rosea, Gliocladium catenulatum, T. atroviride, T. harzianum*, and *U. novo-zealandiae* have already been used for developing commercial fungicides against *B. cinerea* [4]. For example, *G. roseum* is a fungus that has demonstrated its ability to limit the growth of *B. cinerea* by limiting the availability of nutrients through competition for their acquisition, either in the phylloplane or in the senescent leaves. [189].

Finally, competition, for both space and nutrients, is considered the main mode of action in yeasts [65,195]. Competition for space is not very specific, as it involves the inhibition of fungal growth in general rather than inhibiting the growth of particular species [196]. This is enhanced by the ability to form a biofilm, which confers some advantages, like the wound protection produced by *Pichia angusta* ANY-67 biofilms, caused by *Botrytis* infection [191]. Regarding nutrients, yeasts can compete for iron, methionine, leucine, and other nutrients [65] that are needed for several fungal processes, like the germination of fungal spores [195]. Some examples of *Botrytis* biocontrol, *Rhodotorula glutinis* F147 and *Cryptococcus albidus* F131, compete for nutrients that are needed for conidial germination in *Botrytis* [192]. Some yeasts have also been studied with regard to competition for biocontrol in post-harvest conditions, for example, *Wickerhamomyces anomalus* YE06, which has been shown to compete with *B. cinerea* in cherry tomatoes for both space and nutrients [193].

### 5.2. Induction of Systemic Resistance

Plants possess their own defense system that includes physical, chemical, and induced defenses [182]. The latter is defined as “the process of active resistance dependent on the host plant’s physical or chemical barriers, activated by biotic or abiotic inducing agents” [186]. This means that its activation depends on some trigger, which can be biotic or abiotic [197]. The pathogen itself may be the trigger that activates this resistance through biochemical reactions or production of pathogenic proteins [103]. For example, during infection, *B. cinerea* produces polygalacturonases, which hydrolyze plant cell wall components, releasing oligogalacturonides that induce a variety of host defense responses [198]. In addition, some non-pathogenic microorganisms can also activate induced resistance [14]. Induced resistance has been shown to be efficient for vegetative tissue biocontrol, and it can be applied locally or systematically [58].

Induced resistance is usually divided in two main groups, systemic acquired resistance (SAR) and inducible systemic resistance (ISR) [182]. The former can be defined as the inherent immunity of the plant, and it is activated either by direct exposure to biotic triggers, both pathogens and non-pathogens, or by abiotic factors, including a number of chemical compounds [1,199]. This mechanism mainly depends on the accumulation of pathogenesis-related proteins and the production of salicylic acid [200], a chemical compound that is usually produced after infection [2]. For example, endophytic microorganisms, such as *Pseudomonas aeruginosa* 7NSK2, *P. fluorescens* CHA0, *P. aeruginosa* 7NSK2, and *Serratia marcescens* 90–166, produce salicylic acid, which induces resistance against *B. cinerea* in beans and enhances host defense [186,187]. Salicylic acid production is also induced by the production of VOCs like trichodiene, produced by *T. arundinaceum* IBT 40837, which provides plant defense against *Botrytis* [99]. However, little is known about the resistance against *B. cinerea* induced by SAR [199].

The latter is similar to a hypersensitive response resulting from the exposure of PGP microorganisms [200]. Here, ethylene and jasmonic acid are the two phytohormones that induce the response [2]. Both rhizobacteria and bacterial endophytes have been demonstrated to induce ISR. For example, *Burkholderia phytofirmans* PsJN, a plant endophyte, induces accumulation of phenolic compounds and strengthens the plant during the colonization of cell walls in the exodermis against *B. cinerea* on grapevines [183]. In the same way, *B. subtilis* FB17 can induce ISR in plants via stimulation of the jasmonic and ethylene pathways [182]. There are also studies on the specific bacterial metabolites that induce ISR. In this sense, *B. velezensis* Bvel1 produces azelaic acid, which triggers the host immune response in *A. thaliana* [73]. Fungi like *Trichoderma* have also been studied for their ability to induce ISR. Thus, *T. harzianum* in tobacco confers resistance to *B. cinerea* through the expression of L-amino acid oxidase since this enzyme activates the expression of defense-related genes and genes involved in salicylic acid, jasmonic acid, and ethylene biosynthesis [201]. Molecules produced by *Trichoderma*, such as Th-LAAO, have also been studied because they activate plant defense mechanisms, contributing to the ISR of the host against *B. cinerea* [190]. Finally, some yeasts are also related to the induction of resistance. For example, *Candida saitoana* 240, *C. oleophila* 182, and *Metschnikowia* NRRL Y-30752 enhance the innate immune response in plants, increasing pathogen resistance [65].

## 6. Conclusions

Fungi, specifically *B. cinerea*, are some of the main threats to the agriculture industry. They are not only responsible for 40% of crop losses worldwide but also for economic losses of USD 10 to 100 billion. Until now, chemical pesticides have been the most useful solution to this problem; however, they come with negative environmental consequences that are now rejected by the population. This, together with the specter of resistance, has led to the suggestion that a more sustainable and safer alternative is needed. In this respect, biopesticides have been studied for the last few years, and they were proven to be an innovative solution. Their potential lies in the use of PGP microorganisms, which are known to possess different plant growth promotion and fungal biocontrol mechanisms, providing a wide variety of new sources for the development of biopesticides. The wide diversity of microorganisms and microbial metabolisms has revealed a great number of modes of action with antifungal potential. In this review, we highlighted some of the most studied mechanisms. One of the most studied is the production of secondary metabolites, where siderophores (e.g., pyoverdine, enterochelin, bacillibactin), lytic enzymes (e.g., chitinase, endoglucanase, protease), antibiotic substances (e.g., surfactin, fengycin, gliotoxin), and volatile organic compounds (VOCs) (e.g., benzothiazole, trichodermol, 3-methyl-1-butanol) are included. Their production depends on the type of microorganism involved, but we can confirm some genera that are outlined among the others, such as *Bacillus*, *Pseudomonas,* and *Trichoderma*. They produce a great variability of secondary metabolites with antifungal activity, like cyclic lipopeptides in *Bacillus*, siderophores in *Pseudomonas*, and lytic enzymes in *Trichoderma*.

Additionally, other mechanisms have been classified as protection mechanisms, with competition and induction of systemic resistance being the most important, and other microorganisms have also been studied in recent years, for example, *Metschnikowia pulcherrima* and *Serratia plymuthica*.

Although their effectiveness in vitro has been tested against several phytopathogens, including *B. cinerea*, further research needs to be done in order to elucidate how they work and what their effects are when applied in the field. Fortunately, the wide diversity of molecules resulting from the wide diversity of microbial metabolisms is a powerful weapon in the fight against plant fungal diseases since it may affect different targets in the *Botrytis* life cycle and structure. Some of the most studied have already been tested, and they are currently being used in the agriculture industry with satisfactory results.

## Figures and Tables

**Table 1:** Overview of literature reporting the inhibition of *Botrytis cinerea* through the production of diffusible antifungal metabolites.

Antifungal Microorganisms	Condition/Plant	Antifungal Metabolite	Antifungal Effect	Reference
**Bacteria**				
*Actinoalloteichus cyanogriseus* 12A22	In vitro	2-Hydroxyethyl-3-methyl-1,4-naphthoquinone	Growth inhibition	[70]
*Bacillus amyloliquefaciens* VB7	In vitro and foliar application	Phthalic acid, hept-3-yl isobutyl ester and propanoic acid,2-hydroxy-, methyl ester	Conidia parasitationSuppression of mycelial growth	[71]
*Bacillus subtilis* NCD-2	Apple fruit	Fengycin	Open pores in the plasma membrane	[72]
*Bacillus velezensis* Bvel1	Pepper and grape plants	Bacillibactin	Suppression of fungal growth by chelating the available ferric iron	[73]
*Bacillus velezensis* XT1	In vitro and in fruits (Tomatoes, grapes, strawberries)	Surfactin, fengycin, and bacillomycin	Open pores in the plasma membrane	[56]
*Kosakonia radicincitans* DSM 16656	In vitro and in apple fruit	Enterochelin	Blocking the polygalacturonase	[74]
*Ochrobactrum cicero* MM17	In vitro and *Lilium* L.	Propanoic acid, −hydoxy-methyl ester; phthalic acid, hex-3-yl isobutyl ester and phthalic acid, hept-3-yl isobutyl ester	Suppression of mycelial growth	[75]
*Pantoea* sp. MQT16M1	In vitro and in strawberry fruits	Salicylamide, maculosin, and herniarin	Disruption of cell wall components	[76]
*Paraburkholderia phytofirmans* PsJN (*Pseudomonas* sp. PsJN)	In vitro	Lipopolysaccharides	Cytoplasm coagulation	[71]
*Pseudomonas aeruginosa* 7NSK2	Tomato plants and *Arabidopsis thaliana*	Pyochelin and pyocyanin	Induction of systemic resistance (ISR)	[77]
*Pseudomonas fluorescens* fp-5	Strawberry plants	Hydroxamate-type siderophores	Prevention of plant infection	[78]
*Pseudomonas* sp. CHA0	Soil	Pyoverdine or pseudobactin	Iron depletion	[50]
*Rahnella aquatilis* BNM	In vitro	Enterochelin	Blockage of polygalacturonase	[79]
**Fungi**				
*Acremonium persicinum* MF-347833	In vitro	VL-2397 (cyclic hexapeptide)	Suppression of hyphal elongation	[80]
*Aspergillus fumigatus* AF293	In vitro	Gliotoxin	DNA disruption	[81]
*Gliocladium virens* 41	In vitro	Gliotoxin	Inhibition of spore germination	[82]
*Trichoderma atroviride* SJ3-4	In vitro and in *Phaseolus vulgaris* cv. Borlotto seeds	Glucose oxidase dermadin, trichovirdin and sesquiterpene, heptalic acid	Effect on spore germination	[83]
*Trichoderma* sp. BV1	*Rubus* sp.	Gliotoxin	Suppression of conidial germination	[84]
**Yeasts**				
*Aureobasidium pullulans* L47	Post-harvest grape berries, kiwi fruit, and strawberries	Aureobasidins	Inhibition of inositol phosphoryl ceramide synthase	[85]
*Metschnikowia pulcherrima* MPR3	In vitro	Pulcherrimin	Iron sequestration	[64]
*Pichia membranifaciens* CYC 1106	Apple fruits	Killer toxin	Damage on membrane, glucanase activity, inhibition of β-1,3-glucansynthase, cell cycle arrestation, and inhibition of calcium uptake	[86]
*Rhodotorula glutinis* ySL 30	In vitro	Rhodotorulic acid	Inhibition of polygalacturonase and laccase	[87]
*Saccharomyces cerevisiae* CBS8112	Post-harvest pears	Killer toxin	Damage to the membrane, glucanase activity, inhibition of β-1,3-glucansynthase, cell cycle arrestation, and inhibition of calcium uptake	[88]

**Table 2:** Overview of literature reporting the inhibition of *Botrytis cinerea* by volatile antifungal metabolites production.

Antifungal Microorganisms	Condition/Plant	Antifungal Metabolite	Antifungal Effect	Reference
**Bacteria**				
*Bacillus amyloliquefaciens* VB7	In vitro and foliar application	Phthalic acid, hept-3-yl isobutyl ester and propanoic acid,2-hydroxy-, methyl ester	Conidia parasitation Suppression of mycelial growth	[98]
*Bacillus nakamurai* TR2, *Bacillus pseudomycoides* DHT2, *Bacillus proteolyticus* H2F1 and *Bacillus thuringiensis* H1R2	In vitro and in organic tomato fruits	3-methylbutan-1-ol, sulfur-containing compounds, 2-heptanone, and dodecanal	Suppression of mycelial growth	[139]
*Bacillus velezensis* ZSY-1	In vitro	Pyrazine [2,5-dimethyl], benzothiazole, phenol (4-chloro-3-methyl), and phenol-2,4-bis (1,1-dimethylethyl)	Suppression of mycelial growth and sporulation	[138]
*Lactiplantibacillus plantarum* UFG 121	In vitro and in kiwifruits	Phenyllactic acid and 4-hydroxyphenyllactic acid	Suppression of mycelial growth	[147]
*Pantoea* sp. MQT16M1	Grapevine plants	Phenylethyl alcohol	Reduction of the length of internal necrosis	[76]
*Pseudomonas aeruginosa* LV	In vitro	Phenazine-1-carboxylic acid	Suppression of mycelial growth	[142]
*Pseudomonas chlororaphis* ZL3	In vitro and in Chinese cherry	1-dodecene and dimethyl disulfide	Reduction of disease incidence and lesion diameter	[140]
*Pseudomonas fluorescens* ZX	In vivo and in grapes	Dimethyl trisulfide and geranyl formate	Suppression of mycelial growth and spore germination. Reduction of disease incidence and the disease index	[143]
*Pseudomonas protegens* CHAo	In vitro	Ammonia and dimethyl trisulfide	Suppression of mycelial growth	[141]
*Streptomyces* sp. S97	In vitro and in strawberries	3-carene 2,5-dione, geosmin, beta-cubebene, and one phenolic compound	Inhibition of Botrytis cinerea decay on strawberries and suppression of germination	[144]
**Fungi**				
*Hypoxylon* sp. CI-4	In vitro	1,8-cineole, 1-methyl-1,4-cyclohexadiene	Suppression of mycelial growth	[149]
*Metarhizium anisopliae* Ma70	In vitro and in apple fruits	1-octanol	Suppression of conidia germination and mycelium growth	[150]
*Phomopsis* sp. By 254	In vitro	Sabinene, 1-butanol, 3-methyl; benzene ethanol; 1-propanol, 2-methyl, and 2-propanone	Suppression of mycelial growth	[152]
*Trichoderma* spp.	In vitro	Trichodermol, harzianum A, and harzianolide	Suppression of mycelial growth	[101]
**Yeasts**				
*Aureobasidium pullulans* EXF-6519	In vitro and in tomato fruits and grapes	3-methyl-1-butanol	Suppression of mycelial growth and reduction of fungal incidence	[153]
*Candida intermedia* C410	In vitro and in strawberry fruits	1, 3, 5, 7-cyclooctatetraene, 3-methyl- 1-butanol, 2-nonanone, and phenylethyl alcohol	Suppression spore germination and mycelial growth	[155]
*Scheffersomyces spartinae* W9, *Candida pseudolambica* W16	In vitro and in strawberry fruits in planta	Unknown	Suppression of mycelial growth and reduction of disease incidence in fruits	[156]
*Sporidiobolus pararoseus* YCXT3	In vitro and in strawberry fruits	2-ethyl-1-hexanol	Suppression of spore germination and mycelial growth	[154]

**Table 3:** Overview of literature reporting the inhibition of *Botrytis cinerea* through hydrolytic enzyme production.

Antifungal Microorganisms	Condition/Plant	Antifungal Metabolite	Antifungal Effect	Reference
**Bacteria**				
*Bacillus amyloliquefaciens* Y1	In vitro	β-1,3-glucanase	Suppression of mycelial growth. Modification of the hyphal structure	[165]
*Bacillus halotolerans* KLBC XJ-5	In vitro and in strawberry fruits	Chitinase and β-1, 3-glucanase	Suppression of mycelial growth and reduction of conidial germination	[166]
*Bacillus subtilis* KLBC BS6	In vitro and in blueberry fruits	Chitinase	Suppression of mycelial growth and reduction of conidial germination	[167]
*Paenibacillus xylanexedens* Z2–4	In vitro	Chitinase	Suppression of mycelial growth	[168]
*Pseudomonas elgii* HOA73	In vitro	Chitinase	Suppression of spore germination	[34]
*Serratia plymuthica* C48	In vitro	Chitinase	Suppression of spore germination and germ-tube elongation	[169]
*Virgibacillus marismortui* M3-23, *Terribacillus halophilus* J31, *Halomonas elongate* L80, *Planococcus rifietoensis* M2-26, *Staphylococcus equorum* B1-35 and *Staphylococcus* sp. J23	In vitro and in strawberry fruits	Chitinase, β-1,3-glucanase, cellulase and protease	Reduction of gray mold rot incidence and fungal growth	[170]
**Fungi**				
*Albifimbria verrucaria* SYE-1	In vitro and in grape leaves	Chitinase	Suppression of conidium germination and mycelial growth	[171]
*Gliocladium roseum* Bainier	In vitro	β l-3 glucanase	Breakdown of glucans in hyphal walls	[172]
*Gliocladium virens* 41	In vitro	Endochitinase	Suppression of spore germination and hyphal elongation	[173]
*Trichoderma harzianum* NCIM1185	Bean leaves	Extracellular proteases	Inactivation of fungal hydrolytic enzymes and attack of fungal cell wall components	[174]
*Trichoderma harzianum* T39	Bean leaves	Exo- and endo-polygalacturonase, pectin methyl esterase, pectate lyase, cutinase, chitinase, and β-1,3-glucanase	Reduction of spore germination	[175]
**Yeasts**				
*Aureobasidium pullulans* PI1	In vitro and in grape and mandarin fruits	β-1,3-glucanase, pectinase, and protease	Disruption of cell wall components and competition for nutrients	[63]
*Aureobasidium pullulans* PL5	In vitro	Alkaline serine protease	Reduction of spore germination and germ-tube length	[176]
*Galactomyces candidum* JYC1146	In vitro and in strawberry fruits	Chitinase	Control of fungal growth and reduction of disease severity	[19]
*Pichia anomala* K	Apple fruits	Exo-β-glucanases	Disruption of cell wall components	[177]
*Cryptococcus laurentii* LS28	Apple fruits	β-1,3-glucanase	Reduction of disease appearance in postharvest conditions	[178]

**Table 4:** Overview of literature reporting the inhibition of *Botrytis cinerea* by other mechanisms.

Antifungal Microorganisms	Condition/Plant	Antifungal Metabolite	Antifungal Effect	Reference
**Bacteria**				
*Bacillus amyloliquefaciens* BBC047	Tomato leaves	-	Biofilm formation	[181]
*Bacillus subtilis* FB17	Tomato plants	-	Induction of systemic resistance [ISR]	[182]
*Bacillus velezensis* Bvel1	Arabidopsis thaliana	Azelaic acid	Induction of systemic resistance [ISR]	[73]
*Burkholderia phytofirmans* PsJN	Grapevines	H_2_O_2_ production	Induction of systemic resistance [ISR]	[183]
*Lactobacillus plantarum* CM-3	Strawberry wounds	-	Colonization	[184]
*Pantoea ananatis* BLBT1-08	Grapevine leaves	-	Colonization	[185]
*Pseudomonas aeruginosa* 7NSK2, *Pseudomonas fluorescens* CHA0, *Pseudomonas aeruginosa* 7NSK2, and *Serratia marcescens* 90-166	Bean plants	Salicylic acid	Induction of systemic resistance [ISR]	[186,187]
*Pseudomonas syringae* L-59-66	Pear fruits	-	Competition for nutrients	[188]
**Fungi**				
*Aureobasidium pullulans* L1	In vitro and in peach fruits	Hydroxamate-type siderophore	Iron competition	[105]
*Gliocladium roseum*	Strawberry leaves	-	Nutrient competition	[189]
*Trichoderma arundinaceum* IBT 40837	Tomato plants	Trichodiene	Induction of defense-related genes	[99]
*Trichoderma harzianum* Th-LAAO	Tobacco leaves	L-amino acid oxidase	Induction of defense-related genes	[190]
*Trichoderma harzianum* T39 and *Ulocladium atrum* 385	Laboratory conditions	-	Competition for nutrients and colonization of necrotic tissue	[58]
**Yeasts**				
*Pichia angusta* ANY-67	Apple fruit	-	Wound protection	[191]
*Rhodotorula glutinis* F147 and *Cryptococcus albidus* F131	In vitro	-	Competition for iron, methionine, leucine, and other nutrients	[192]
*Candida saitoana* 240, *Candida oleophila* 182, and *Metschnikowia fructicola* NRRL Y-30752	Fruits	Overproduction of reactive oxygen species	Induction of innate immune responses	[65]
*Wickerhamomyces anomalus* YE06	Cherry tomatoes	-	Competition for space and nutrients	[193]
